# Vitamin D-linked vulnerability and functional connectivity alterations in the superior frontal gyrus contributing to cognitive impairment in Parkinson’s disease

**DOI:** 10.3389/fnagi.2025.1657723

**Published:** 2025-11-19

**Authors:** Lingling Lv, Lixia Qin, Shenglan Hu, Changlian Tan, Haiyan Liao, Hainan Zhang, Feng Ren, Chunyu Wang

**Affiliations:** 1 Department of Neurology, The Second Xiangya Hospital, Central South University, Changsha, China; 2 Department of Radiology, The Second Xiangya Hospital, Central South University, Changsha, China; 3 Key Laboratory of Hunan Province in Neurodegenerative Disorders, Central South University, Changsha, China; 4 Department of Geriatric Surgery, The Second Xiangya Hospital, Central South University, Changsha, China; 5 Department of Medical Genetics, The Second Xiangya Hospital, Central South University, Changsha, China; 6 Hunan Province Clinical Medical Research Center for Genetic Birth Defects and Rare Diseases, Department of Medical Genetics, The Second Xiangya Hospital, Central South University, Changsha, China

**Keywords:** Parkinson’s disease, cognitive impairment, vitamin D, gray matter volume, superior frontal gyrus

## Abstract

**Background and aims:**

Forecasting specific factors influencing cognitive impairment (CI) in Parkinson’s disease (PD) patients can improve clinical outcomes. This study aims to identify brain areas vulnerable to vitamin D deficiency and assess functional integrity in PD patients with and without CI.

**Methods:**

Thirty-four PD patients [14 with CI (PD-CI), 20 with normal cognition (PD-NC)] and 21 healthy controls (HCs) underwent serum vitamin D testing, T1-weighted MRI, and resting-state functional MRI (rs-fMRI). Voxel-based morphometry (VBM) was used to compare gray matter volume (GMV) between PD patients and HCs. Whole-brain multiple regression analyses, adjusted for age and sex, identified GMV regions associated with vitamin D levels. Resting-state functional connectivity (FC) analyses were performed using vitamin D-related regions as seeds. Correlation and multivariate regression analyses, adjusted for Hoehn and Yahr stage and age, assessed relationships among FC, cognitive performance, and vitamin D levels.

**Results:**

Compared with HCs, PD patients exhibited significant GMV loss, affecting widespread brain regions including the middle frontal gyrus (MFG), superior frontal gyrus (SFG), and hippocampus. Region of interest (ROI)-based analysis revealed that vitamin D levels were associated with GMV in the bilateral MFG and SFG (*r* = −0.406, *p* = 0.021). These findings suggest that the MFG and SFG are vulnerable regions in PD patients linked to vitamin D levels. To assess the impact of abnormal vitamin D levels on relevant resting-state networks, clusters encompassing the bilateral SFG were used as ROIs. The intrinsic connectivity network of the vulnerable area, using the bilateral SFG as seed regions, revealed abnormal functional connectivity with several brain networks, including the visual network, the default mode network, the executive control network, the sensorimotor network, and the memory network. Abnormal FC values within the SFG functional network were associated with disease severity, cognitive dysfunction, and vitamin D levels (*p* < 0.05). Multi-model regression analyses revealed that connectivity in the left SFGmed network was negatively associated with CI in PD, with vitamin D levels showing a potential protective effect.

**Conclusion:**

The SFG is associated with vitamin D levels in PD patients, and disruptions in its structural and functional connectivity may link to CI. Future longitudinal studies are necessary to confirm these associations and explore the potential impact of vitamin D supplementation on cognitive function in PD.

## 1 Introduction

Parkinson’s disease (PD) is a prevalent neurodegenerative disorder characterized by the degeneration and loss of dopaminergic neurons in the substantia nigra and the formation of Lewy bodies within neurons (Bloem et al., 2021). Although PD is primarily recognized for its motor symptoms, it also encompasses a wide range of non-motor symptoms, including cognitive impairment (CI), which can emerge even in the early stages of the disease. The incidence of PD-CI is significantly higher in individuals with PD, ranging from two to six times that of the healthy population. Both the cumulative incidence and prevalence of PD-CI increase as the disease progresses (Maetzler et al., 2009). This cognitive decline not only severely impacts the quality of life of patients but also significantly increases the burden on caregivers (Aarsland et al., 2021; Marinus et al., 2018).

These epidemiological observations highlight the critical need for a comprehensive understanding of PD-CI, including its etiology and the mechanisms underlying cognitive decline. In recent years, there has been growing recognition of the role of vitamin D in the pathophysiology of various neurodegenerative diseases, including PD, Alzheimer’s disease (AD), and multiple sclerosis (MS) (Janjusevic et al., 2022). Preliminary evidence indicates that imaging, neurophysiology, and peripheral biomarkers hold promise for the diagnosis and prognosis of PD-CI (Svenningsson et al., 2012). Notably, advancements in neuroimaging techniques have enhanced the ability to detect pathological changes, which may facilitate the early identification and management of cognitive impairment in PD. These developments underscore the importance of continued research to elucidate the pathophysiological processes and improve clinical outcomes for individuals with PD-CI.

Studies utilizing structural and functional neuroimaging techniques have identified *in vivo* imaging markers linked to the development of CI in PD. Previous research has demonstrated that dementia in PD is associated with extensive gray matter (GM) atrophy, resembling the patterns observed in AD. This atrophy is particularly notable in the parietal–temporal lobe, entorhinal cortex, hippocampus, prefrontal cortex, and posterior cingulate cortex (Svenningsson et al., 2012). In addition to these structural changes, worsening cognitive function in PD has been correlated with alterations in functional networks, as assessed by resting-state fMRI (rs-fMRI). These alterations include changes in functional connectivity (FC) within regions of the default-mode network (DMN) (Lucas-Jimenez et al., 2016; Disbrow et al., 2014). However, despite the associations between functional networks, GM atrophy, and CI, the specific factors influencing the vulnerability of these regions and their roles in the progression from PD to PD with dementia (PDD) remain unclear.

Vitamin D is a fat-soluble hormone capable of crossing the blood–brain barrier, exerting neuroprotective and nutritional effects on the central nervous system (Tuckey et al., 2019). The role of vitamin D in neurodegenerative diseases has gained significant attention, as it is known to exert neuroprotective effects by influencing synaptic plasticity, reducing inflammation, and modulating neurotrophic factors (Pertile et al., 2018; Mayne and Burne, 2019; Lima et al., 2018). A Mendelian randomization study suggests a causal relationship between vitamin D and cognitive performance in older adults (Li et al., 2024). Notably, vitamin D plays essential roles in synaptic plasticity and cognitive processes (Mayne and Burne, 2019). Neuropsychological testing has revealed a significant positive correlation between vitamin D levels and cognitive function in patients with PD (Peterson et al., 2013), although the underlying pathophysiological mechanisms remain unclear. In cases of AD and dementia, neuroimaging studies have shown that vitamin D deficiency impacts cognitive function by altering the GMV of brain structures such as the thalamus, cingulate cortex, hippocampus, and temporal gyrus, as well as the integrity of white matter structures (Moon et al., 2015; Ali et al., 2020; Hooshmand et al., 2014). Furthermore, vitamin D insufficiency has been linked to a thinner cingulate cortex and precuneus cortex in older community-dwellers (Foucault et al., 2019; Annweiler et al., 2014). However, the role of vitamin D in brain function changes among patients with PD-CI has not been previously investigated. Given the heterogeneity of cognitive profiles in PD compared to AD, which predominantly includes deficits in attention-executive functions rather than the typical visuospatial function and memory impairments seen in AD, understanding the specific impact of vitamin D on cognitive decline in PD is essential (Bronnick et al., 2011; Kehagia et al., 2010). Therefore, vitamin D deficiency may contribute to cognitive decline independently of PD, or the interplay between the two mechanisms could accelerate cognitive impairment. Investigating the specific relationships between vitamin D and cognitive impairment in PD remains a crucial area of research.

In this study, we hypothesized that vitamin D may influence cognitive function by affecting the integrity of brain structures and function in brain regions related to cognitive function in PD. To test this hypothesis, we utilized voxel-based morphometry (VBM) and resting-state functional MRI (rs-fMRI) to evaluate the effects of vitamin D on brain morphology and functional network alterations in PD patients, both with and without CI. More specifically, leveraging cross-sectional assessments of cognitive abilities and neuroimaging biomarkers in PD patients, we aim to: (1) evaluate the differences in vitamin D levels and GMV changes between PD patients and healthy controls (HCs); (2) investigate the vulnerable brain areas and associated functional networks that are highly sensitive to vitamin D; and (3) assess the relationships between CI and the structural and functional integrity of networks in these vulnerable areas. Through these aims, we seek to advance our understanding of the role of vitamin D in cognitive function among PD patients and to uncover new avenues for therapeutic intervention.

## 2 Materials and methods

### 2.1 Study design and participants

**Figure 1:**
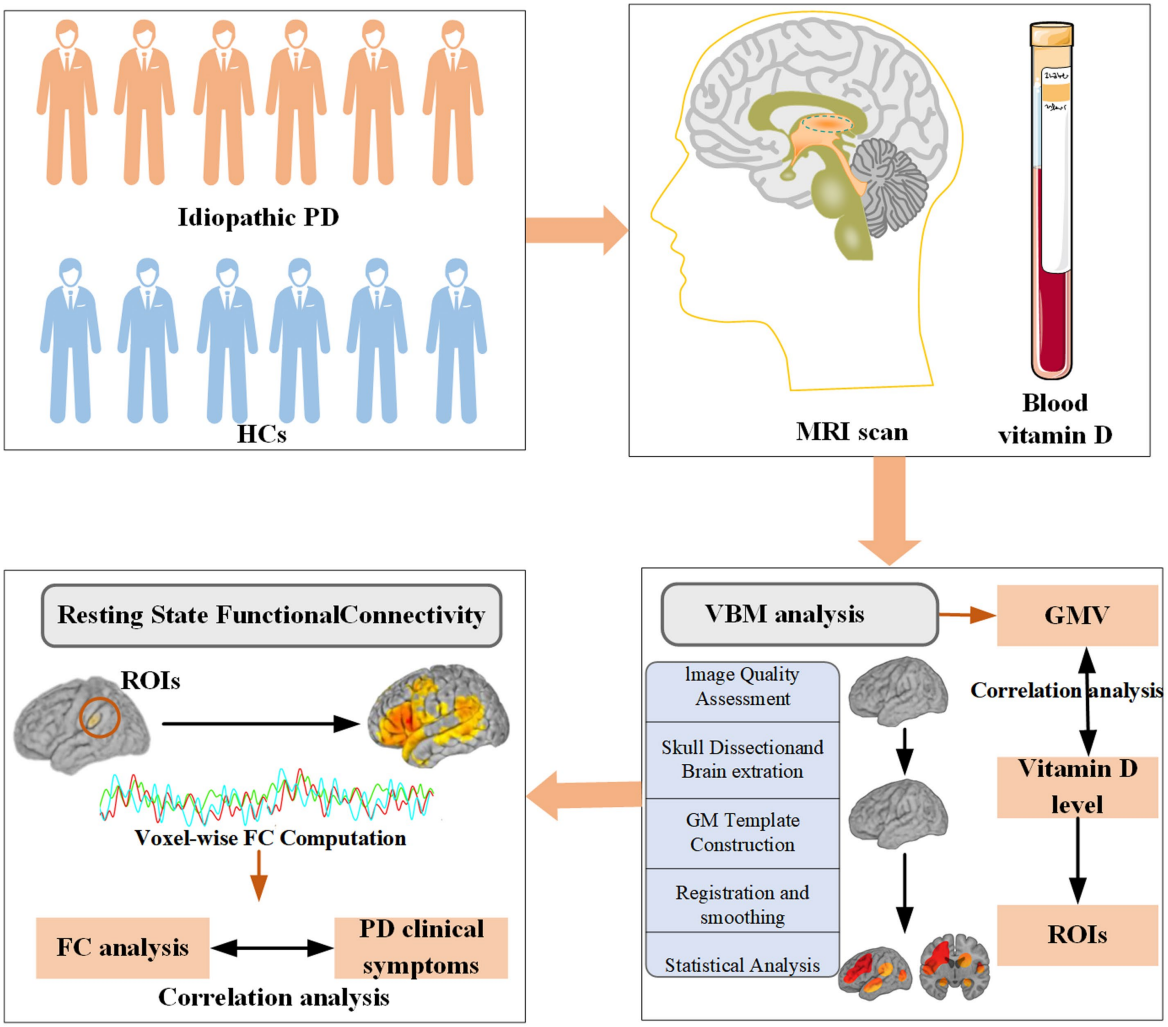
Study procedures: Each PD patient was examined by a neurologist using a standardized and structured interview. Sociodemographic characteristics, clinical features of PD, and assessments of motor and nonmotor symptoms were recorded. Both HCs and PD patients were included for gray matter and FC analysis. PD, Parkinson’s disease; HCs, healthy controls; FC, functional connectivity; ROIs, regions of interest; GMV, gray matter volume.

Patients were consecutively recruited throughout the year from outpatient clinics specializing in movement disorders at the Second Xiangya Hospital of Central South University. Before analysis, data completeness was assessed, and participants with missing MRI, serum 25-hydroxyvitamin D, or questionnaire data were excluded from the corresponding analyses. Because the proportion of missing data was minimal (<5%), no data imputation was performed. In the end, 34 patients with idiopathic PD were included in this cross-sectional study. All included patients met the movement disorder society (MDS) clinical diagnostic criteria for PD (Postuma et al., 2015). Patients were followed for a minimum of 12 months, with the diagnosis reassessed and confirmed at the end of the follow-up period. Exclusion criteria at enrollment included the diagnosis of secondary Parkinson-plus syndromes, parkinsonism, gene-related PD, use of vitamin D supplements, history of any chronic disease, or the use of medications that could potentially impact the absorption of vitamin D. Additionally, 21 healthy controls (HCs), matched to PD patients for age, sex, and years of education, were recruited from volunteers and the spouses of PD patients. All controls were cognitively normal, with no symptoms of PD or history of neurological diseases. Controls were excluded if they had a first- or second-degree relative with PD, and the remaining exclusion criteria were the same as those for the PD group. The relatively small sample size was mainly due to the strict inclusion criteria and the availability of eligible PD patients who completed all imaging and serum assessments. Considering the exploratory nature of this study and our earlier research, which reported a medium sized sample linking vitamin D to PD (PD: *N* = 330, HC: *N* = 209) (Lv et al., 2021), the current sample size is considered sufficient to potentially detect meaningful group differences with reasonable statistical power. The study procedures are described in Figure 1. The study was approved by the ethics committee of the Second Xiangya Hospital of Central South University. Informed written consent was obtained from all subjects prior to their inclusion in the study.

### 2.2 Assessments and statistical analysis

The demographic characteristics of HCs and PD patients, including age, age of onset, sex, years of education, height, and weight, were collected. Clinical characteristics for all PD patients were assessed, encompassing disease duration, drug history, the Unified Parkinson’s Disease Rating Scale (UPDRS II, III, IV, and total), the modified Hoehn and Yahr Staging Scale (H&Y stage), the Parkinson’s Disease Questionnaire (PDQ39), the Mini-Mental State Examination (MMSE). Based on MMSE scores, patients were categorized into PD with normal cognition (PD-NC) and PD-CI. Following criteria established by previous studies (Zhang et al., 1990; Katzman et al., 1988; Marder, 2010; Williams-Gray et al., 2006), MMSE scores were classified as follows: ≤17 for illiterate subjects, ≤20 for grade-school literate, and ≤23 for those with junior high school education or higher.

The acquisition of blood samples and rs-fMRI data was performed on the same day as the neuropsychological testing. Serum 25-hydroxyvitamin D [25(OH)D] levels were measured using liquid chromatography-mass spectrometry (LC-MS). To minimize potential confounders affecting serum vitamin D levels, we adopted the same methodological controls as our previous study (Lv et al., 2021). Specifically, patients who used vitamin D supplements, had chronic illnesses, or were taking medications that could influence vitamin D absorption were excluded. Participants were recruited continuously throughout the year, ensuring a balanced distribution across different months, thereby minimizing the confounding effect of seasonal variation in vitamin D levels. As demonstrated in our previous work (Lv et al., 2021), this recruitment strategy effectively eliminated the seasonal bias in serum vitamin D measurements. All evaluations for PD patients, including MRI studies and neuropsychological tests, were conducted in the OFF-medication state. This OFF-medication state was achieved by the withdrawal of dopaminergic medications 12 to 18 h prior to testing.

The statistical analyses were conducted using the Statistical Package for Social Sciences (SPSS), version 26 (IBM SPSS Statistics 26). For continuous data that met the criteria for normal distribution and homogeneity of variance, differences between two groups were assessed using the independent samples *t*-test, while differences among three or more groups were evaluated using analysis of variance (ANOVA). In cases where the data did not meet these criteria, the Mann–Whitney *U* test and Kruskal–Wallis test were employed for two-group and multi-group comparisons, respectively. Categorical variables were analyzed using the *χ*^2^ test or Fisher’s exact test, as appropriate. Correlation analyses were performed using multiple regression analysis, controlling for age and gender, to identify the association between MMSE scores and vitamin D levels in patients with PD. Statistical significance was defined as *p* < 0.05.

### 2.3 MRI data acquisition

The MRI data was acquired using a Siemens MAGNETOM Skyra 3T MRI scanner. Functional images were obtained through Echo Planar Imaging (EPI) sequences with the following parameters: repetition time (TR) = 2,500 ms, echo time (TE) = 25 ms, flip angle (FA) = 90°, matrix size = 64 × 64, field of view (FOV) = 240 mm × 240 mm, slice thickness = 3.5 mm, and 39 slices with no gap between slices during the resting condition. Additionally, axial T1 structural images were acquired using T1-weighted imaging (T1WI) three-dimensional magnetization-prepared rapid acquisition gradient echo (3D MP-RAGE) sequences. The specific parameters for these sequences were: TR = 1,900 ms, TE = 2.01 ms, FA = 9°, slice thickness = 1 mm, 176 slices, FOV = 256 mm × 256 mm, and a matrix size of 256 × 256. During the MRI scans, subjects were instructed to keep their eyes closed while avoiding falling asleep. To minimize head motion and reduce noise, foam pads were used to stabilize the head.

### 2.4 Voxel-based morphometry analysis

VBM analysis was conducted using FMRIB’s FSL software suite.^1^ The methodological steps are detailed as follows: (1) Image quality assessment: The T1-weighted images for each subject were visually inspected to ensure there were no significant quality issues; (2) Skull dissection and brain extraction: Each subject’s T1 image underwent skull stripping to remove non-brain tissues, focusing on isolating the brain matter; (3) GM Template construction: (1) Segmentation: Post skull stripping, each subject’s image was segmented into GM, white matter (WM), and cerebrospinal fluid (CSF); (2) Initial template formation: The GM images were initially aligned to the Montreal Neurological Institute (MNI152) standard space using linear registration to create a preliminary GM template; (3) Final template refinement: These GM images were then non-linearly registered to the preliminary GM template, resulting in the final group-specific GM template. (4) Registration and smoothing: All individual GM images were registered to the group-specific GM template obtained in the previous step. The registered images were then smoothed at varying kernel sizes to produce t-value maps with different levels of smoothness. (5) Statistical analysis and multiple comparison correction: Group comparisons were conducted using the “randomize” tool in FSL, employing a non-parametric permutation approach. Each comparison involved 5,000 permutations to ensure robust statistical analysis and correction for multiple comparisons.

After pre-processing, to account for potential confounding factors, age and sex were included as nuisance variables in the comparisons between PD subgroups and HCs identified through cluster analysis. Significant clusters were identified using the threshold-free cluster enhancement (TFCE) method (Smith and Nichols, 2009) with statistical significance defined as *p* < 0.05. The results of the group comparisons were visualized using FSLview. To pinpoint the vulnerable brain regions with GM that are highly sensitive to vitamin D levels, we conducted a Kendall’s tau-b correlation analysis. This analysis controlled for age and gender, aiming to explore the relationship between GMV and vitamin D levels.

### 2.5 Rs-fMRI data analysis

DPABI (Yan et al., 2016) (http://rfmri.org/DPABI; version, V5.1_201201), Data Processing Assistant for Resting-State fMRI (DPARSF 5.1), SPM12^2^ and REST_V1.8_130615 (Harrison et al., 2011) software based on Matlab R2019B are used to analysis rs-fMRI data (Yan et al., 2016; Chao-Gan and Yu-Feng, 2010). The standard processing steps in DPARSF included: (1) convert data from DICOM format to NIFTI format; (2) remove the first 10 time points; (3) time and space correction; (4) head motion correction; (5) normalize by using DARTEL toolbox; (6) smooth: spatial smoothing with an isotropic 6-mm full width at half maximum (FWHM) Gaussian kernel; (7) after normalization and smoothing, the waveform of each voxel was finally used for removal of the linear trends of time courses and for temporal band-pass filtering (0.01 to 0.08 Hz).

### 2.6 Calculate functional connectivity

For the network construction of vulnerable areas associated with vitamin D, we selected vulnerable GM regions as seeds to perform voxel-wise FC analyses for PD sub-groups and HCs. The voxel-wise FC analyses were conducted by computing the temporal correlations between the mean time series of each seed region and the time series of each voxel across the whole brain. This process yielded correlation coefficients (r values), which were subsequently transformed into *Z* values using Fisher’s *r*-to-*z* transformation to ensure that the correlation coefficients followed a normal distribution. These *Z* values were then used to calculate the *T* values for the FC strength between the regions of interest (ROIs) and other brain regions throughout the whole brain (Chao-Gan and Yu-Feng, 2010). For the FC maps, we performed a two-tailed one-sample *t*-test on the *Z* maps. In the FC analysis, the AlphaSim method was employed to correct for multiple comparisons. The significance threshold was set at a *p*-value of <0.005 before correction and <0.05 after correction. Additionally, analyses were performed to examine the correlations between FC and disease severity, as well as cognitive impairment. We further performed binary logistic regression analysis, adjusting for age and H&Y stage, to investigate the impact of SFG FC on cognitive impairment in PD. Prior to model estimation, the variance inflation factor (VIF) was used to assess multicollinearity, and predictors with a VIF greater than 10 were excluded. For each model, odds ratios (OR) with 95% confidence intervals were calculated. To adjust for bias due to small sample size and extreme data, we further applied Firth logistic regression using the profile penalized log-likelihood method in RStudio to explore the effect of SFG FC on cognitive impairment in PD patients. All analyses were performed in both SPSS (Version 27.0) and RStudio (Copyright^©^ 2025 by Posit Software, PBC). The threshold for statistical significance was established at *p* < 0.05.

## 3 Results

### 3.1 Clinical characteristics and vitamin D levels among groups

The demographic and clinical characteristics, as well as vitamin D levels, of the 34 PD patients and 21 HCs are summarized in Table 1. No significant differences were found between the HC group and the PD subgroup in terms of age, BMI, and sex. Additionally, there were no statistically significant differences between the PD-CI and PD-NC group regarding disease duration, UPDRS scores (II, III, IV, and total), Hoehn and Yahr stage, Levodopa Equivalent Daily Dose (LEDD), and Parkinson’s Disease Questionnaire (PDQ-39) scores (*p* > 0.05). The onset age of patients in the PD-CI group was older than that of patients in the PD + NC group. Furthermore, the PD-CI subgroup had significantly fewer years of education compared to the PD-NC subgroup [6 (5, 9) vs. 10 (9, 12); Bonferroni corrected *p* = 0.002]. The PD-CI subgroup also exhibited lower MMSE scores. No significant differences were observed in the levels of 25(OH)D_3_ between the two subgroups. Similarly, there were no significant differences in the levels of 25(OH)D, although the levels of 25(OH)D were lower in the PD-CI group compared to the PD-NC group, which may be related to the small sample size.

**Table 1:** Demographic characteristics of the PD patients and healthy controls.

Variable	HC (*N* = 21)	PD (*N* = 34)		Degrees of freedom	*χ*^2^/*F*/*U*/*K* values	*p*-value
		PD-NC (*n* = 20)	PD-CI (*n* = 14)			
Sex^a^, male/female	12/9	14/6	6/8	2	2.509	0.285
BMI^b^		23.67 ± 3.24	21.63 ± 2.69	32	0.534	0.062
Age, y^d^	56 (52, 61)	55 (51, 57)	63 (56, 71)	2	5.062	0.080
Onset age, y^b^	NA	52 ± 6	60 ± 12	32	7.876	0.017
Education, y^d^	10 (9, 12)	8 (6, 11)	6 (5, 9)^e^	2	10.262	0.006
PD duration, m^c^	NA	18 (12, 54)	18 (5, 36)	33	119.5	0.467
UPDRS-I^c^	NA	2 (1, 3)	3 (2, 4)	33	69	0.011
UPDRS-II^c^	NA	10 (7, 12)	12 (7, 16)	33	109	0.276
UPDRS-III^c^	NA	14 (11, 25)	19 (13, 38)	33	108	0.262
UPDRS total^c^	NA	24 (19, 38)	34 (22, 56)	33	105	0.220
H&Y stage^c^	NA	1.5 (1, 2.3)	2 (1.5, 2.5)	33	111.5	0.306
25(OH)D_3_^b^		22.42 ± 7.73	20.42 ± 5.24	32	2.884	0.408
25(OH)D^b^		22.55 ± 7.61	20.90 ± 5.54	32	1.757	0.495
LEDD, mg/d^c^	NA	131.3 (0, 425)	0 (0, 237.5)	33	109	0.240
PDQ-39^c^	NA	18 (11, 24)	32 (14, 50)	33	95.5	0.119
MMSE^d^	29 (29, 30)	26 (25, 28)	19 (14, 21)	2	41.760	0.000

BMI, body mass index; PD, Parkinson’s disease; PD-NC, Parkinson’s disease with normal cognition; PD-CI, Parkinson’s disease with cognitive impairment; LEDD, levodopa dosage; H&Y stage, Hoehn & Yahr stage; PDQ-39, 39-item Parkinson’s Disease Questionnaire 39; MMSE, Mini-Mental Status Examination; UPDRS, the Unified Parkinson’s Disease Rating Scale; NA, not applicable.

a *χ*^2^ statistics.

b Two student’s *t*-test.

c Mann–Whitney *U* test.

d Kruskal–Wallis test.

e Comparison of education in PD-CI patients and HC with Bonferroni correction, *p* = 0.004.

### 3.2 Changes of gray matter volume in PD groups

Compared to the HCs, individuals with PD exhibited notable atrophy in both the cerebral cortex and cerebral white matter. Moreover, the PD cohort demonstrated widespread GMV loss across the entire brain. Specifically, notable abnormalities were detected in several regions, including the middle frontal gyrus (MFG), superior frontal gyrus (SFG), hippocampus, left thalamus, middle temporal gyrus, cingulate gyrus, frontal pole, angular gyrus, postcentral gyrus, supramarginal gyrus, precentral gyrus, caudate, temporal pole, and cerebellum (right VI) (TFCE corrected *p* < 0.05). Supplementary Figure 1 provides a visual representation of these results.

### 3.3 Vulnerable areas associated with vitamin D levels

**Figure 2:**
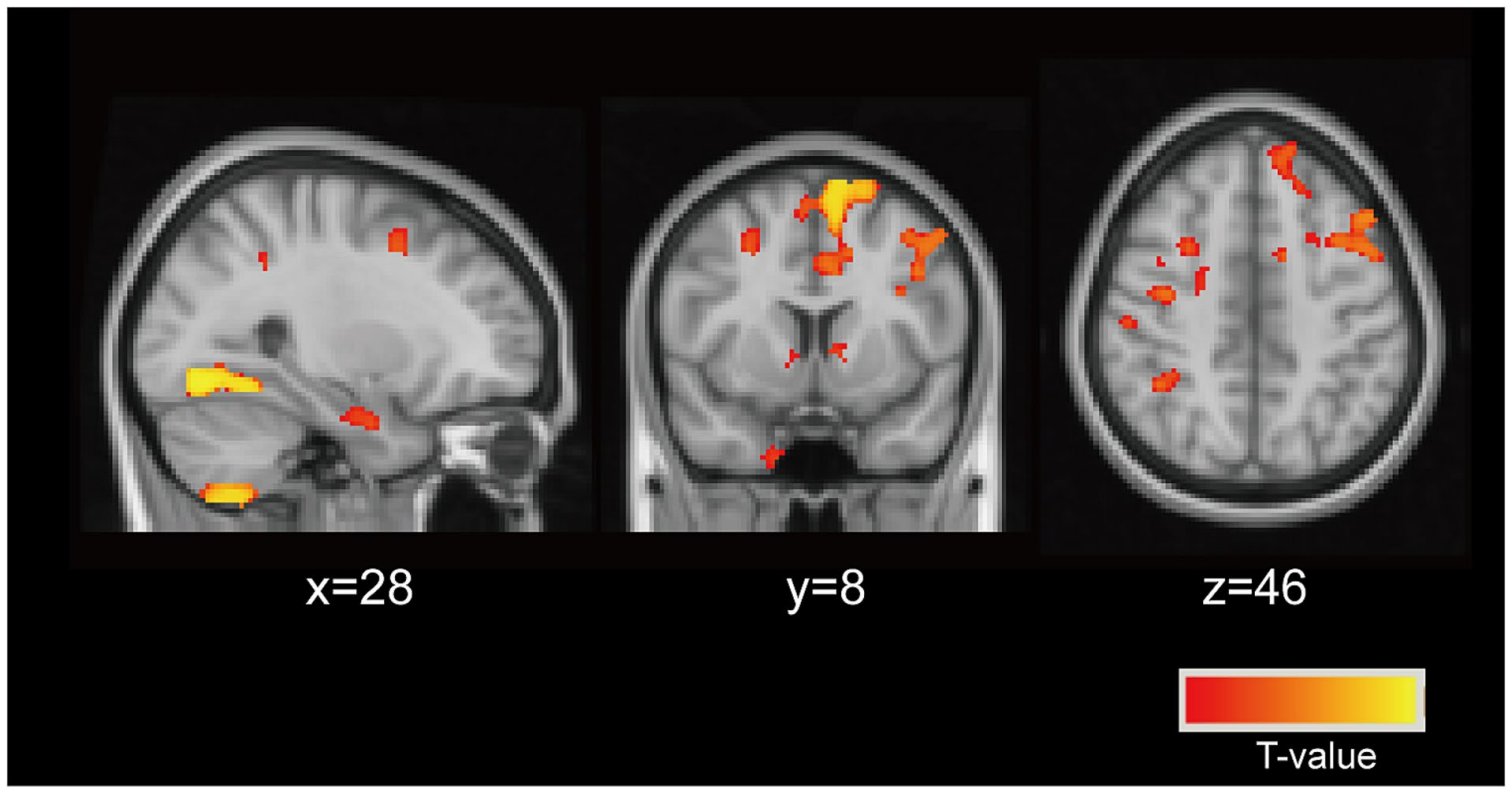
Neuronal vulnerable areas associated with vitamin D levels. The particular voxel located at coordinates (MIN, *x* = 28, *y* = 8, *z* = 46) were identified as the vulnerable areas associated with vitamin D levels in PD patients (controlling for age, and sex). This voxel predominantly encompassed the bilateral middle frontal gyrus (MFG), superior frontal gyrus (SFG).

In this study, we identified brain regions where GMV differed between PD patients and HCs, defining these areas as ROIs. Using ROI-based Kendall’s tau-b correlation analysis, while controlling for age and gender, we sought to identify specific GMV regions in PD patients associated with vitamin D levels. Our analysis revealed a significant association between 25(OH)D levels and a particular voxel (see Figure 2, TFCE corrected *p* < 0.05, cluster > 112) located at coordinates (MIN, *x* = 28, *y* = 8, *z* = 46) (*r* = −0.406, corrected *p* = 0.021). This voxel predominantly encompassed the bilateral MFG and SFG. These findings establish the MFG and SFG as vulnerable regions in PD patients linked to vitamin D levels.

### 3.4 Analysis of resting-state functional connectivity networks connected to the vulnerable areas

In the VBM analysis, GMV differences between PD patients and HCs were more pronounced in the SFG compared to the MFG (see Figure 2). Furthermore, we analyzed whole-brain functional connectivity (FC) patterns across the HC, PD-NC, and PD-CI groups using MFG as the ROI and found no significant association between cognitive function and the MFG functional network (data not provided). To further assess the impact of abnormal vitamin D levels on relevant resting-state networks, we selected clusters encompassing the bilateral SFG, specifically the medial SFG (SFGmed) and medial orbital SFG (SFGmorb) regions, as four ROIs. These regions were used to analyze FC patterns across the entire brain among the three groups. Spherical seed regions with a radius of 2 mm were created based on the AAL3 template (Rolls et al., 2020) within MATLAB R2019B software.

#### 3.4.1 Left SFGmed FC

**Figure 3:**
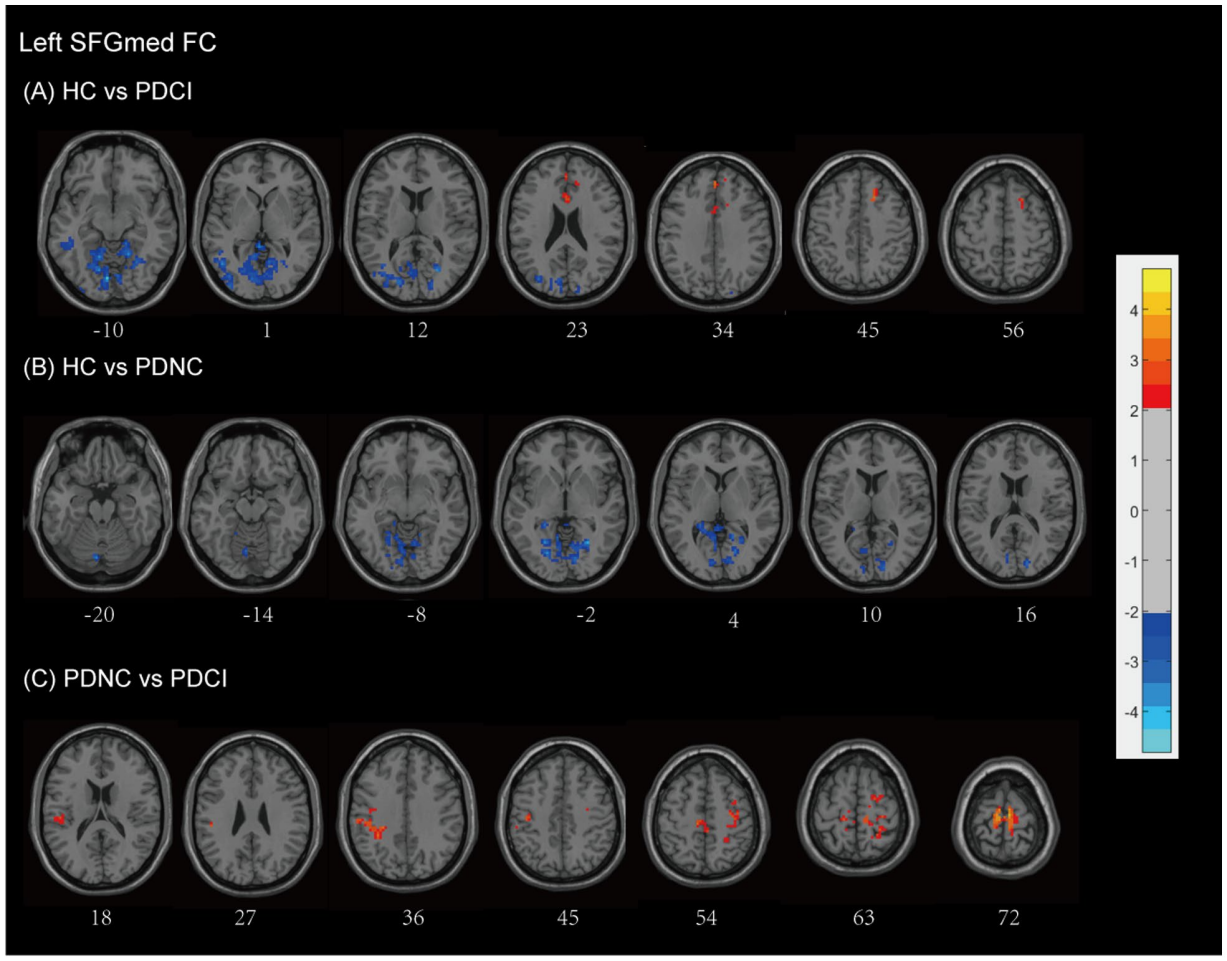
Left SFGmed functional network differences between the subgroups of PD patients and healthy controls. **(A)** PD-CI vs. HC: Increased functional connectivity (FC) in occipital, temporal, cerebellar, and limbic regions (blue); decreased FC in middle frontal and cingulate areas (red). **(B)** PD-NC vs. HC: Increased FC in occipital and limbic regions (blue). **(C)** PD-CI vs. PD-NC: Decreased FC in frontal–parietal sensorimotor areas (red). All findings are corrected for multiple comparisons, at AlphaSim, *p* < 0.05. Color bar represents *T*-values; coordinates are in MNI space. PD-NC, Parkinson’s disease with normal cognition; PD-CI, Parkinson’s disease with cognitive impairment; HC, healthy control; SFGmed, the medial of superior frontal gyrus.

The connectivity of the left SFGmed in patients PD-CI exhibited significant differences compared to the HC group (see Figure 3A and Table 2). Patients with PD-CI exhibited increased FC between the left SFGmed and multiple posterior cortical and limbic regions, including the occipital lobe (middle and superior occipital gyrus, lingual gyrus, cuneus, and calcarine cortex), temporal lobe (middle temporal gyrus and fusiform gyrus), limbic lobe (posterior cingulate, parahippocampal gyrus), cerebellum anterior lobe, and Brodmann areas 18 and 19. Conversely, reduced FC was observed with the middle frontal gyrus (MFG), and limbic lobe (cingulate gyrus and cingulum) (AlphaSim corrected, cluster >153 voxels), suggesting weakened fronto-limbic connectivity.

**Table 2:** Group differences in resting-state functional connectivity among PDCI, PDNC, and HC.

Comparison	Cluster	Brain region (AAL3)	Cluster size (voxels)	Peak MNI coordinates			*T*-value (peak intensity)	Effect
				*x*	*y*	*z*		
Seed1(SFGmed.L)								
HC vs. PDCI	Cluster 1	Cerebellum anterior lobe, the occipital lobe, the temporal lobe, the limbic lobe, and Brodmann areas 18 and 19	1,229	−3	−78	−12	−4.8253	Increased FC in PDCI
	Cluster 2	Middle frontal gyrus, and limbic lobe	179	0	42	30	4.8141	Decreased FC in PDCI
HC vs. PDNC	Cluster 1	Cerebellum anterior lobe, culmen, occipital lobe, limbic lobe, and Brodmann area 18	524	27	−60	−3	−3.9599	Increased FC in PDNC
PDNC vs. PDCI	Cluster 1	Parietal lobe, frontal lobe	173	−39	−36	39	3.3435	Decreased FC in PDCI
	Cluster 2	Parietal lobe, frontal lobe, and Brodmann area 6	368	9	−30	69	4.5561	Decreased FC in PDCI
Seed2(SFGmed.R)								
HC vs. PDCI	Cluster 1	Left temporal lobe and occipital lobe, and Brodmann areas 18 and 19	560	−36	−90	18	−4.2531	Increased FC in PDCI
	Cluster 2	Right occipital lobe	245	42	−87	−6	−4.5341	Increased FC in PDCI
HC vs. PDNC	Cluster 1	Anterior lobe of the cerebellum, culmen, occipital lobe, temporal lobe, limbic lobe, and Brodmann areas 18, 19, and 30	703	57	−57	9	−4.102	Increased FC in PDNC
PDNC vs. PDCI	Cluster 1	Right frontal lobe and parietal lobe, and Brodmann area 6	181	30	−12	48	3.78	Decreased FC in PDCI
	Cluster 2	Left frontal lobe and parietal lobe, and cingulate gyrus	386	12	−24	69	4.9714	Decreased FC in PDCI
Seed3(SFGmorb.L)								
HC vs. PDCI	Cluster 1	Midbrain, pons, lentiform nucleus, putamen, pallidum, insula, caudate, thalamus, ventral lateral nucleus, frontal lobe, and limbic lobe	868	9	−15	−9	4.4277	Decreased FC in PDCI
	Cluster 2	Occipital lobe, temporal lobe, limbic lobe, parietal lobe, and Brodmann area 18,17 and 19	1,332	−27	−72	−3	−4.6473	Increased FC in PDCI
HC vs. PDNC	Cluster 1	Lentiform nucleus, putamen, midbrain, thalamus, brainstem, pallidum, lateral globus pallidus, caudate, frontal lobe, limbic lobe, temporal lobe, and Brodmann area 13	899	12	33	−6	4.9369	Decreased FC in PDNC
PDNC vs. PDCI	Cluster 1	Frontal lobe, paracentral lobule, postcentral gyrus, and Brodmann area 6	172	15	−24	66	4.6698	Decreased FC in PDCI
Seed4(SFGmorb.R)								
HC vs. PDCI	Cluster 1	Midbrain, brainstem, temporal lobe, rontal lobe, limbic lobe, thalamus, lentiform nucleus, rolandic operculum, putamen, and Brodmann areas 11 and 13	847	−3	−24	−24	4.8888	Decreased FC in PDCI
	Cluster 2	Frontal lobe, limbic lobe, thalamus, lentiform nucleus, and Brodmann area 11	350	3	15	21	4.3295	Decreased FC in PDCI
	Cluster 3	Cerebellum anterior lobe, occipital lobe, temporal lobe, limbic lobe, and Brodmann areas 17 and 19	1,536	−33	−78	24	−5.2549	Increased FC in PDCI
HC vs. PDNC	Cluster 1	Cerebellar posterior lobe, cerebellar tonsil, pons, brainstem	339	18	−63	−36	4.2117	Decreased FC in PDNC
	Cluster 2	Cerebellum, brainstem, putamen, thalamus, caudate, lentiform nucleus, lateral globus pallidus, rolandic operculum, ventral lateral nucleus, temporal lobe, limbic lobe, frontal lobe, and Brodmann areas 13, 10, and 47	1,315	36	15	15	3.9165	Decreased FC in PDNC
	Cluster 3	Right frontal lobe, and Brodmann area 6	198	24	−6	63	−3.6336	Increased FC in PDNC
PDNC vs. PDCI	Cluster 1	Right occipital lobe, temporal lobe	197	21	−84	−6	−3.5315	Increased FC in PDCI
	Cluster 2	Left occipital lobe, temporal lobe, and inferior frontal gyrus	258	−24	−81	6	−4.2337	Increased FC in PDCI

Positive peak intensity values indicate reduced functional connectivity (FC) in the second group of the comparison (e.g., “HC vs. PDCI” → decreased FC in PDCI). Negative peak intensity values indicate increased FC in the second group (e.g., “HC vs. PDCI” → increased FC in PDCI). Cluster locations are reported in MNI coordinates and identified using the AAL3 atlas. PD-NC, Parkinson’s disease with normal cognition; PD-CI, Parkinson’s disease with cognitive impairment; HC, healthy control; L, left; R, right; SFG, superior frontal gyrus; SFGmed, the medial of superior frontal gyrus; SFGmorb, medial orbital of superior frontal gyrus; MNI, Montreal Neurological Institute.

Similarly, PD-NC patients demonstrated enhanced SFGmed FC relative to HCs (see Figure 3B and Table 2), involving the cerebellar regions (anterior lobe, culmen), occipital lobe (lingual gyrus, cuneus, calcarine cortex), limbic lobe (posterior cingulate), and Brodmann area 18 (AlphaSim corrected, cluster >158 voxels), consistent with activation in visual-associative and cerebellar networks.

When comparing PD-CI to PD-NC patients, the PD-CI patients exhibited reduced FC between the left SFGmed and regions spanning the frontal lobe (precentral gyrus, MFG, supplementary motor area), parietal lobe (inferior parietal gyrus, postcentral gyrus, inferior parietal lobule, supramarginal gyrus, paracentral lobule), and Brodmann area 6, with AlphaSim correction and a cluster size threshold of >163 voxels (see Figure 3C and Table 2). These findings indicate a loss of sensorimotor and executive network integration associated with cognitive impairment in PD.

#### 3.4.2 Right SFGmed FC

**Figure 4:**
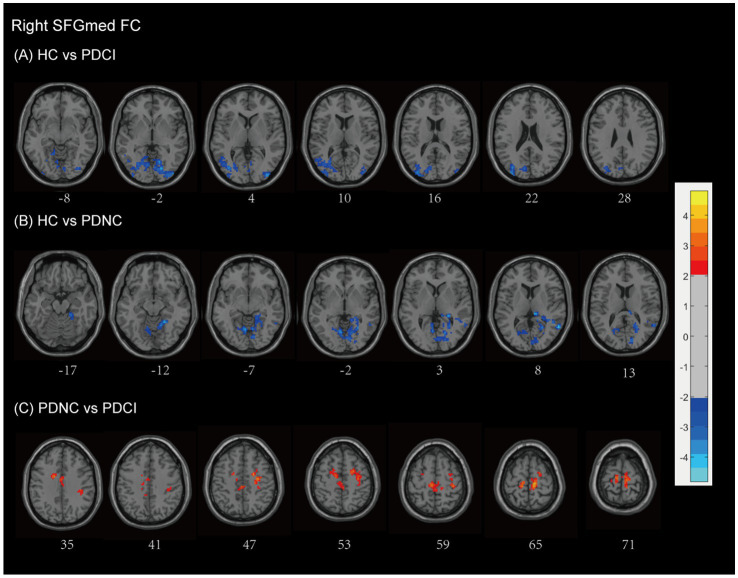
Right SFGmed functional network differences between the subgroups of PD patients and healthy controls. **(A)** PD-CI vs. HC: Increased functional connectivity (FC) in the occipital lobe and temporal lobe (blue). **(B)** PD-NC vs. HC: Increased FC in occipital the occipital lobe, temporal lobe, and limbic areas (blue). **(C)** PD-CI vs. PD-NC: Decreased FC in frontal–parietal–cingulate areas (red). All findings are corrected for multiple comparisons, at AlphaSim, *p* < 0.05. Color bar represents *T*-values; coordinates are in MNI space. PD-NC, Parkinson’s disease with normal cognition; PD-CI, Parkinson’s disease with cognitive impairment; HC, healthy control; SFGmed, the medial of superior frontal gyrus.

Using the right SFGmed as a seed, we observed that patients with PD-CI exhibited higher FC levels compared to HCs (see Figure 4A and Table 2). Patients with PD-CI demonstrated increased FC between the right SFGmed and several posterior cortical regions, including the temporal lobe (middle temporal gyrus), occipital lobe (lingual gyrus, cuneus, superior, inferior, and middle occipital gyri), and Brodmann areas 18 and 19 (AlphaSim corrected, with a cluster size >161 voxels).

Similarly, PD-NC also showed elevated FC compared to HCs (Figure 4B and Table 2), particularly involving the occipital lobe (lingual gyrus, calcarine fissure, and surrounding cortex, cuneus), temporal lobe (middle and superior temporal gyri), and the limbic lobe (parahippocampal gyrus, posterior cingulate, hippocampus), as well as the anterior lobe of the cerebellum, culmen, and Brodmann areas 18, 19, and 30 (AlphaSim corrected, with a cluster size >160 voxels).

In contrast, PD-CI patients demonstrated reduced right SFGmed FC relative to PD-NC, mainly within frontal–parietal–cingulate networks, including the MFG, precentral gyrus, supplementary motor area, postcentral gyrus, paracentral lobule, cingulate gyrus (including the cingulum), and Brodmann area 6 (AlphaSim corrected, with a cluster size >170 voxels) (see Figure 4C and Table 2). These findings indicate reduced sensorimotor and executive integration in PD-CI compared to PD-NC.

#### 3.4.3 Left SFGmorb FC

**Figure 5:**
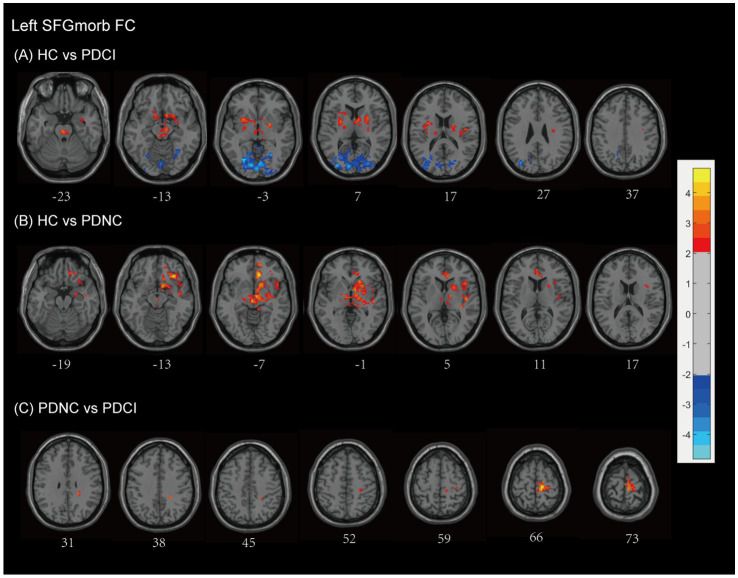
Left SFGmorb functional network differences between the subgroups of PD patients and healthy controls. **(A)** PD-CI vs. HC: Decreased functional connectivity (FC) was mainly observed in subcortical and frontal–limbic regions (red), increased FC was found with posterior cortical areas, mainly in the occipital, temporal, parietal (blue). **(B)** PD-NC vs. HC: Decreased FC primarily in fronto-striatal and limbic circuits (red). **(C)** PD-CI vs. PD-NC: Further FC reductions in precentral, medial frontal, and paracentral regions (red). All findings are corrected for multiple comparisons, at AlphaSim, *p* < 0.05. Color bar represents *T*-values; coordinates are in MNI space. PD-NC, Parkinson’s disease with normal cognition; PD-CI, Parkinson’s disease with cognitive impairment; HC, healthy control; SFGmorb, medial orbital of superior frontal gyrus.

Compared with HCs, patients with PD-CI showed widespread alterations in FC with the left SFGmorb (see Figure 5A and Table 2). Decreased FC was mainly observed in subcortical and frontal–limbic regions, including the basal ganglia (putamen, pallidum, caudate, lentiform nucleus), thalamus, midbrain, pons, insula, subcallosal gyrus, and limbic lobe (parahippocampal gyrus, amygdala), indicating disruption of cortico–subcortical and limbic connectivity. Conversely, increased FC was found with posterior cortical areas, notably the occipital (cuneus, lingual gyrus, middle occipital gyrus, calcarine, superior occipital gyrus), temporal (middle temporal gyrus, fusiform gyrus), limbic lobe (posterior cingulate), parietal (precuneus), and Brodmann areas 18, 17, and 19, with AlphaSim correction applied and a cluster size threshold of >180 voxels.

In PD-NC patients, reduced FC was also observed relative to HCs (see Figure 5B and Table 2), primarily in fronto-striatal and limbic circuits, including the inferior and middle frontal gyri, lentiform nucleus, lateral globus pallidus, caudate, putamen, midbrain, pallidum, anterior cingulate and paracingulate gyri, cingulum, superior temporal gyrus, and Brodmann area 13, with AlphaSim correction and a cluster size threshold of >169 voxels.

When comparing PD-CI with PD-NC, patients with cognitive impairment showed further decreases in FC between the left SFGmorb and the frontal lobe (precentral gyrus, medial frontal gyrus), paracentral lobule, postcentral gyrus, and Brodmann area 6, reflecting additional disruption of motor-related frontal networks. All findings survived AlphaSim correction with a cluster size threshold > 165 voxels (see Figure 5C and Table 2).

#### 3.4.4 Right SFGmorb FC

**Figure 6:**
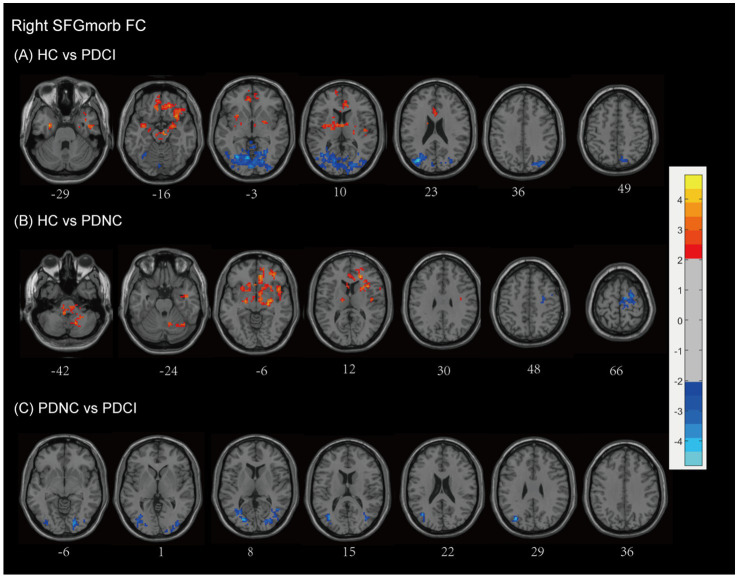
Right SFGmorb functional network differences between the subgroups of PD patients and healthy controls. **(A)** PD-CI vs. HC: Decreased functional connectivity (FC) in frontal–subcortical and limbic regions (red); increased FC in occipital lobe and temporal lobe, parietal lobe, posterior cingulate (blue). **(B)** PD-NC vs. HC: Decreased FC in fronto–striato–limbic and cerebellar networks (red); increased FC in motor-related frontal regions (blue). **(C)** PD-CI vs. PD-NC: PD-CI patients showed higher FC with several posterior and temporal regions (blue). All findings are corrected for multiple comparisons, at AlphaSim, *p* < 0.05. Color bar represents *T*-values; coordinates are in MNI space. PD-NC, Parkinson’s disease with normal cognition; PD-CI, Parkinson’s disease with cognitive impairment; HC, healthy control; SFGmorb, medial orbital of superior frontal gyrus.

Compared with HCs, patients with PD-CI exhibited widespread reductions in FC with the right SFGmorb (see Figure 6A and Table 2). These decreases were primarily located in temporal–frontal, frontal–subcortical and limbic circuits, including the temporal cortex (superior temporal gyrus, inferior temporal gyrus, temporal pole, fusiform gyrus), inferior frontal gyrus, gyrus rectus, corpus callosum, parahippocampal gyrus, anterior cingulate & paracingulate gyri, thalamus, lentiform nucleus, rolandic operculum, putamen, amygdala, ventral anterior nucleus, midbrain, brainstem, and Brodmann areas 11 and 13. Conversely, increased FC was found in posterior cortical and cerebellar regions, including the occipital lobe (lingual gyrus, middle, superior and inferior occipital gyrus, cuneus, calcarine fissure and surrounding cortex), temporal lobe (middle temporal gyrus and fusiform gyrus), parietal lobe (precuneus), limbic lobe (posterior cingulate), cerebellum anterior lobe, and Brodmann areas 17 and 19, possibly reflecting compensatory visual–associative network engagement (AlphaSim corrected, cluster size >173 voxels).

PD-NC patients also showed decreased FC relative to HCs, mainly involving fronto–striato–limbic and cerebellar regions, including the frontal lobe (inferior frontal gyrus, MFG, corpus callosum, and subcallosal gyrus), temporal lobe (superior temporal gyrus), pons, putamen, thalamus, caudate, lentiform nucleus, lateral globus pallidus, rolandic operculum, ventral lateral nucleus, limbic lobe (anterior cingulate & paracingulate gyri), cerebellar posterior lobe, cerebellar tonsil, and Brodmann areas 13, 10, and 47. However, However, increased FC was observed in motor-related frontal regions, notably the MFG, precentral gyrus, and supplementary motor area, and Brodmann area 6 (AlphaSim corrected, cluster size >171 voxels) (see Figure 6B and Table 2).

When directly comparing PD-CI and PD-NC groups, PD-CI patients demonstrated higher FC between the right SFGmorb and several posterior and temporal regions, including the middle occipital gyrus, lingual gyrus, and cuneus, middle temporal gyrus, and inferior frontal gyrus (AlphaSim corrected, cluster size >163 voxels) (see Figure 6C and Table 2). These results suggest altered integration between prefrontal and posterior cortical areas associated with cognitive decline in PD.

In general, the intrinsic connectivity network of the vulnerable area, using bilateral SFG as seed regions, demonstrated abnormal FC with several brain networks. Specifically, the vulnerable areas showed connectivity with the visual network (including regions such as the cuneus, superior occipital gyrus, fusiform gyrus, Brodmann area 19, middle occipital gyrus, and lingual gyrus), the default mode network (DMN) (comprising the middle temporal gyrus, parahippocampal gyrus, cingulate gyrus, cingulum, medial frontal gyrus, and precuneus), the executive control network (including the MFG, cingulate gyrus, cingulum, and cingulum bundle), the sensorimotor network (consisting of the precentral gyrus, supplementary motor area, postcentral gyrus, paracentral lobule, and Brodmann area 6), and the memory network (notably the parahippocampal gyrus). These patterns indicate widespread disruptions in the connectivity between the vulnerable areas and networks involved in visual processing, executive control, motor functions, and memory, particularly in patients with cognitive impairment.

### 3.5 Relationships among disease severity, cognitive function, vitamin D levels and the SFG functional network

**Figure 7:**
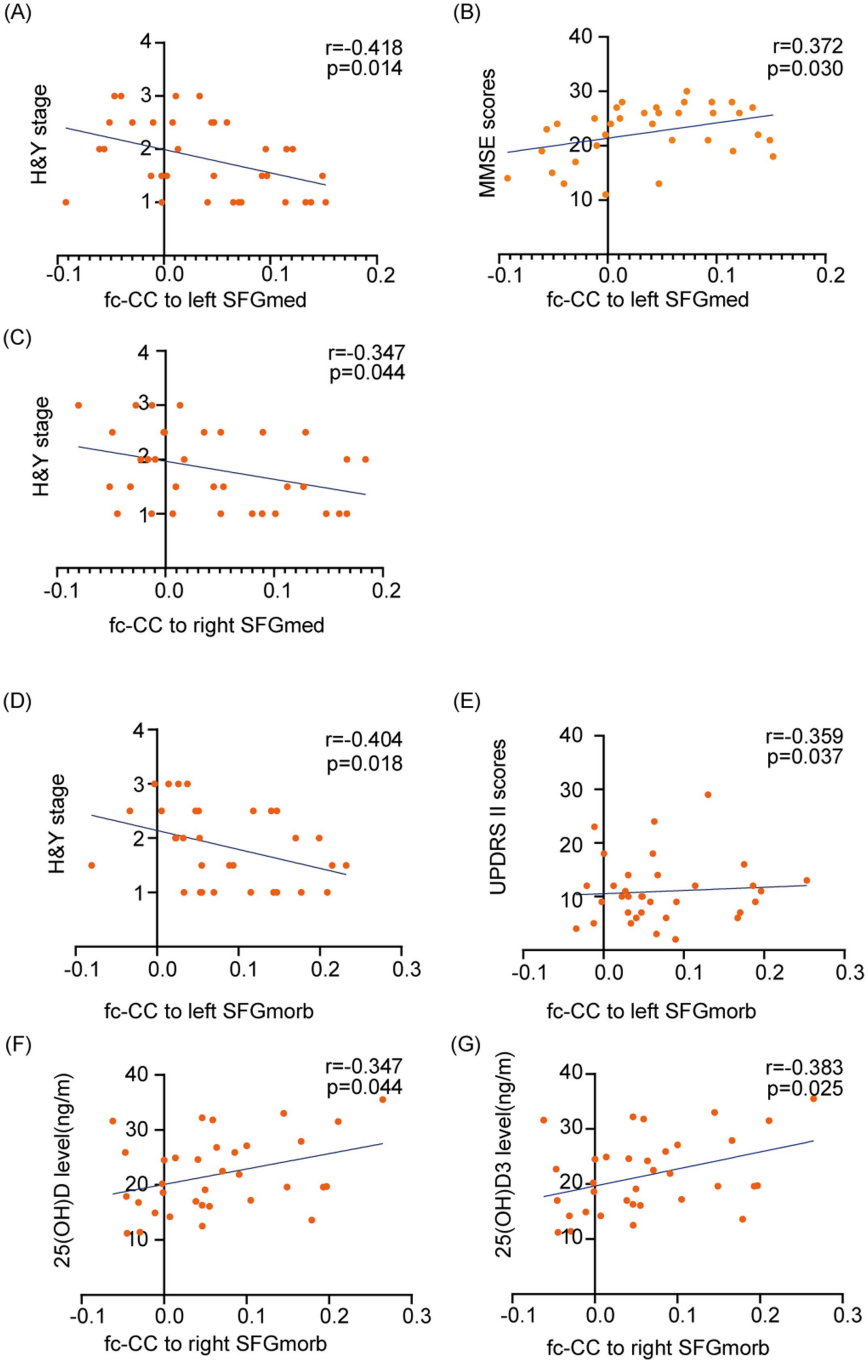
Correlations between disease severity, cognitive function, vitamin D levels, and functional connectivity within the SFG functional network. The fc-CC values within the SFG network are associated with PD clinical symptoms, including Hoehn and Yahr stage, UPDRS II scores, cognitive dysfunction, and vitamin D levels. (**A–C**) Associations between fc-CC values of the left and right SFGmed and H&Y stage or MMSE scores. (**D, E**) Associations between fc-CC values of the left SFGmorb and H&Y stage or UPDRS II scores. (**F, G**) Associations between fc-CC values of the right SFGmorb and serum 25(OH)D and 25(OH)D₃ levels. Each dot represents one PD participant; lines indicate linear regression fits. Pearson’s r and p-values are shown for each correlation. SFGmed, Medial of superior frontal gyrus; SFGmorb - Medial orbital of superior frontal gyrus.

In this study, correlation analyses were conducted to explore the interrelations among disease severity, cognitive function, and functional connectivity correlation coefficient (fc-CC) values in patients with PD. Our findings demonstrate that abnormal fc-CC values within the SFG functional network are associated with several factors, including disease severity (Hoehn and Yahr stage), UPDRS II scores, cognitive dysfunction, and vitamin D levels (Pearson correlation analysis, *p* < 0.05) (see Figure 7). Specifically, abnormalities in the fc-CC values of the left SFGmed functional network were significantly correlated with H&Y stage (*r* = −0.418, *p* = 0.014), and MMSE scores (*r* = 0.372, *p* = 0.03). In parallel, anomalies in the fc-CC values of the right SFGmed functional network were linked to H&Y stage (*r* = −0.347, *p* = 0.044). Furthermore, deviations in the fc-CC values of the left SFGmorb functional network were correlated with UPDRS II scores (*r* = −0.359, *p* = 0.037), and H&Y stage (*r* = −0.404, *p* = 0.018). Similarly, irregularities in the fc-CC values of the right SFGmorb functional network were found to be related to vitamin D levels. These findings underscore the complex interplay between neural connectivity and various clinical and cognitive features in PD, highlighting the potential of fc-CC values as biomarkers for disease progression and symptomatology.

### 3.6 Multi-model regression analysis of SFG functional network connectivity and cognitive impairment in PD

Supplementary Table 1 presents the results of a logistic regression analysis assessing the impact of FC in the SFG network and vitamin D levels on cognitive impairment in PD. SFGmed.L Cluster-2 shows a consistent negative association with cognitive impairment across all three models, with a statistically significant regression coefficient in Models 1 (*B* = −27.125, *p* = 0.026), 2 (*B* = −24.253, *p* = 0.040), and 3 (*B* = −22.817, *p* = 0.055), the latter approaching statistical significance. Additionally, in Model 3, 25(OH)D_3_ is significantly associated with cognitive impairment (*B* = −0.173, *p* = 0.047). A trend toward significance is observed for SFGmed.R Cluster-2 (*B* = 0.185, *p* = 0.082), indicating a possible but non-significant positive relationship with cognitive impairment.

Further Firth logistic regression analyses provided additional insights into the relationships between SFG functional network connectivity and cognitive impairment in PD (Table 3). In Model 1, which did not adjust for confounders, connectivity in the left SFGmed network was significantly associated with cognitive impairment, with a negative coefficient suggesting a protective effect (*B* = −21.234, Chisq = 5.456, *p* = 0.020). Model 2, which adjusted for age, confirmed this relationship (*B* = −18.173, Chisq = 4.396, *p* = 0.036). Interestingly, model 3, which further adjusted for H&Y stage, suggested that vitamin D levels might exert a protective effect against cognitive impairment (*B* = −0.123, Chisq = 3.607, *p* = 0.058). In this model, the left SFGmed network showed a trend toward significance (*B* = −16.364, Chisq = 3.607, *p* = 0.058), though it did not reach the conventional threshold for statistical significance. The connectivity of the SFGmorb network, both left and right, showed weaker associations with cognitive impairment.

**Table 3:** Firth logistic regression analysis of the impact of SFG functional network and vitamin D levels on cognitive impairment in PD.

fc-CC values of SFG	*B*	SE	Chisq	*p*-value	95% CI
Model 1					
SFGmed.L Cluster 2	−21.234	10.033	5.456	0.020	−45.710, −3.074
SFGmed.R Cluster 2	12.521	8.847	2.049	0.152	−4.397, 32.631
SFGmorb.R Cluster 1	0.059	10.837	2.849 × 10^−5^	0.996	−22.403, 22.385
SFGmorb.R Cluster 2	4.623	8.907	0.262	0.609	−13.049, 23.622
25(OH)D_3_	−0.094	0.063	2.327	0.127	−0.236, 0.026
Model 2					
SFGmed.L Cluster 2	−18.173	9.310	4.396	0.036	−40.703, −1.098
SFGmed.R Cluster 2	12.573	8.516	2.217	0.137	−3.859, 31.972
SFGmorb.R Cluster 1	1.943	11.300	0.027	0.869	−21.504, 26.601
SFGmorb.R Cluster 2	5.369	9.182	0.323	0.570	−13.179, 25.417
25(OH)D_3_	−0.104	0.062	2.924	0.087	−0.244, 0.015
Age	0.060	0.039	2.429	0.119	−0.015, 0.149
Model 3					
SFGmed.L Cluster 2	−16.364	9.119	3.607	0.058	−38.282, 0.488
SFGmed.R Cluster 2	13.662	8.703	2.516	0.113	−3.092, 33.633
SFGmorb.R Cluster 1	1.557	11.281	0.017	0.896	−22.126, 26.829
SFGmorb.R Cluster 2	6.367	9.252	0.443	0.506	−12.450, 26.812
25(OH)D_3_	−0.123	0.067	3.607	0.058	−0.281, 0.004
Age	0.059	0.037	2.477	0.116	−0.015, 0.141
H&Y stage	0.594	0.613	0.877	0.349	−0.657, 2.027

fc-CC, functional connectivity correlation coefficient; PD, Parkinson’s disease; L, left; R, right; SFG, superior frontal gyrus; SFGmed, the medial of superior frontal gyrus; SFGmorb, medial orbital of superior frontal gyrus.

Importantly, these regression findings are consistent with the group comparison results shown in Table 2. As demonstrated in Table 2, the PD-CI group exhibited lower fc-CC values in the left SFGmed.L Cluster-2 compared with the PD-NC group, indicating reduced functional connectivity in patients with cognitive impairment. Correspondingly, the regression analysis revealed a negative association between SFGmed.L Cluster-2 connectivity and cognitive impairment, suggesting that higher connectivity is linked to a lower risk of cognitive impairment. Together, these findings consistently indicate that the potential of SFG functional network connectivity—particularly within the left SFGmed—is associated with cognitive decline in PD.

## 4 Discussion

This cross-sectional study reports that, in comparing PD patients with healthy controls, PD patients exhibited significantly lower GMV and diffuse atrophy patterns. Notably, we identified the SFG as a particularly vulnerable area, with its atrophy being associated with variations in vitamin D levels. Additionally, we identified a deterioration in the functional network integrity of the SFG, which correlated with the progression of disease severity and cognitive deficits. These associations suggest a link between vitamin D levels and GMV atrophy and functional connectivity alterations in PD-CI patients.

### 4.1 Widespread brain atrophy and neurodegeneration in PD: implications for motor and cognitive decline

Our study revealed significant brain atrophy in individuals with PD, particularly in the cerebral cortex and white matter, along with widespread GMV loss in regions such as the frontal, temporal, and cingulate gyri, hippocampus, thalamus, and cerebellum. These observations are consistent with previous literature (Jia et al., 2015; Zheng et al., 2022; Xu et al., 2020). The findings highlight the extensive neurodegenerative impact of PD, correlating with clinical symptoms and cognitive decline seen in patients (Zhou et al., 2019; Pan et al., 2024; Li et al., 2022). The widespread atrophy and specific regional degeneration emphasize the multifaceted nature of PD, affecting both motor and cognitive domains. Future research should focus on longitudinal studies to track these changes over time and investigate potential interventions to mitigate neurodegeneration. Understanding the precise mechanisms of atrophy in these regions could pave the way for targeted therapies aimed at preserving brain volume and function, ultimately improving the quality of life for individuals with PD.

### 4.2 Association between vitamin D levels and SFG volume in cognitive function of PD patients

This study found a significant correlation between the reduction in GMV of the SFG and vitamin D levels, suggesting that vitamin D levels may be associated with cognitive function in PD patients through their effects on the structure and function of the SFG. The SFG, located on the superior portion of the frontal lobe, is involved in various cognitive functions, including working memory, attention, and executive function management. GMV reduction in the SFG of PD patients may be related to impairments in these cognitive functions. Vitamin D may contribute to this process through its neuroprotective effects, modulating brain regions vital for executive functions.

### 4.3 The role of vitamin D in cognitive function and neuroprotection in PD

Vitamin D is a fat-soluble vitamin known for its role in bone health. Recent research has highlighted its importance in brain function and neuroprotection (Rimmelzwaan et al., 2016; Barichella et al., 2022; Wang et al., 2015). It exerts its action via vitamin D receptors (VDR) present in neurons and glial cells, their expression is maximum in the hippocampus, thalamus, hypothalamus, substantia nigra, orbitofrontal-cortex, cingulate, and amygdala which are the areas essential for cognition and motor control (Moretti et al., 2018; Annweiler et al., 2013; Veenstra et al., 1998). And study demonstrated that VDR polymorphisms were associated with cognitive decline in PD (Gatto et al., 2016). Other studies have shown that low levels of vitamin D are associated with cognitive decline in PD patients (Peterson et al., 2013; Pignolo et al., 2022). The potential benefits of vitamin D for PD patients have also garnered significant attention. Suzuki et al. (2013) conducted a randomized controlled trial and found that PD patients who received daily vitamin D supplementation experienced a stabilization of the severity of PD. However, even less is known about the effects of vitamin D supplementation on cognition in PD patients (Fullard and Duda, 2020). In PD patients, low levels of vitamin D might affect cognitive function through multiple mechanisms. Firstly, vitamin D has anti-inflammatory and antioxidant properties, which can reduce neuroinflammation and oxidative stress, both of which play significant roles in the pathogenesis of PD (Fullard and Duda, 2020; Calvello et al., 2017). Neuroinflammation and oxidative stress, commonly seen in the brains of PD patients, can lead to neuronal damage and death, consequently impairing cognitive functions (Bloem et al., 2021). Secondly, vitamin D can regulate the expression of neurotrophic factors and promote the growth and survival of neurons (Pertile et al., 2018). Thirdly, vitamin D is involved in calcium homeostasis regulation, and calcium signaling is crucial for neurotransmission and synaptic plasticity (Mayne and Burne, 2019; Latimer et al., 2014). While several possible mechanisms exist for the effect of vitamin D on cognitive in PD, the evidence is preliminary, and more study is needed to make any conclusions.

### 4.4 Abnormal functional network connections of the SFG and their role in cognitive deficits in PD patients

Additionally, we also found that the abnormal connections between the SFG and multiple functional networks might contribute to the cognitive deficits observed in PD patients. These findings are consistent with research in both the general population and AD patients, where vitamin D deficiency has been linked to accelerated brain aging and impaired cognitive function (Terock et al., 2022; Sultan, 2022; Shih et al., 2020). Our study further suggests that abnormal fc-CC values within the SFG functional network are associated with PD severity, cognitive dysfunction, and vitamin D levels. Specifically, we found that the abnormal functional network connections between the SFG and several networks—including the visual network, DMN, executive control network, sensorimotor network, and memory network—are characteristic of the PD-CI group. These findings are consistent with our logistic regression analysis, which also highlighted a significant association between functional connectivity in the left SFGmed.L Cluster-2 and cognitive impairment in PD patients.

These results align with previous studies in AD, which have shown that vitamin D deficiency is associated with disruptions in brain connectivity, particularly in the frontal, occipital, and temporal lobes (Fu et al., 2025). Additionally, research has demonstrated a significant correlation between changes in vitamin D levels and volumetric alterations in cortical regions such as the hippocampus, amygdala, and nucleus accumbens in AD (Li et al., 2025). These findings were further corroborated in AD mouse models, where vitamin D supplementation significantly delayed hippocampal and nucleus accumbens atrophy, while also reducing Aβ accumulation (Li et al., 2025). This suggests that vitamin D plays a crucial role in protecting against neurodegeneration and maintaining brain structure and function, as indicated by our findings, suggests a potential protective mechanism that could extend to PD patients. Given the similarities between PD and AD in terms of brain degeneration, future research should investigate whether vitamin D supplementation can help mitigate cognitive decline and brain atrophy in PD, similarly to its effects observed in AD patients.

Building on this, our study highlights the association between vitamin D levels, specific brain networks, and cognitive dysfunction in PD patients. Disruptions in these networks could provide further insights into the mechanisms underlying cognitive decline in PD. These networks are essential for integrating sensory information, maintaining cognitive flexibility, and performing complex tasks. Specifically, the visual network processes visual information, Relatedly, decreased visual performance was associated with cognitive decline and widespread white matter macrostructural changes in PD patients (Zarkali et al., 2021). The DMN remains active during rest and is associated with self-referential and introspective thoughts, Meanwhile, the altered connectivity pattern in the DMN may play a crucial role in the neurophysiological mechanism of cognitive decline in patients with PD (Chen et al., 2022; Zarifkar et al., 2021). The executive control network is responsible for high-order cognitive functions like planning and decision-making, the sensorimotor network involves motor control and sensory processing, and the memory network is responsible for encoding and retrieving memories. Disruptions in these networks can lead to the cognitive deficits observed in PD patients (Dong et al., 2020; Leh et al., 2010; Wang et al., 2021). Abnormal connections between the SFG and these networks might lead to disruptions in information integration and transmission, thereby affecting cognitive function in PD patients. However, due to the cross-sectional nature of this study, it is important to note that the observed relationships between vitamin D levels, SFG atrophy, and cognitive decline are associative and not causal. Longitudinal studies are essential to clarify the temporal dynamics, explore causality, and identify potential confounding factors, such as disease severity, that could influence both vitamin D levels and brain atrophy in PD. Future longitudinal studies are essential to validate these associations. Additionally, research should focus on investigating whether vitamin D supplementation can influence cognitive outcomes and brain atrophy over time in PD patients.

### 4.5 Strengths and limitations

This study has some strengths. Firstly, the association between SFG atrophy and variations in vitamin D levels is particularly intriguing, suggesting a possible nutritional or metabolic influence on brain health in PD patients. This link opens avenues for potential dietary or supplementation interventions that could mitigate or slow the progression of atrophy and cognitive decline. Another strength lies in the study’s examination of functional network integrity. By not only measuring structural changes but also assessing the functional connectivity of the SFG, the research offers a more comprehensive understanding of the brain alterations occurring in PD-CI. This dual approach underscores the complexity of the disease and the interplay between structural and functional brain changes, providing a more holistic view of PD pathology. The correlation of SFG functional deterioration with disease severity and cognitive deficits further strengthens the study. This finding underscores the relevance of the SFG in the clinical progression of PD and suggests that SFG integrity could potentially serve as a biomarker for disease progression and cognitive decline. This potential biomarker utility is valuable for both clinical assessment and the evaluation of therapeutic interventions. Lastly, the study’s consideration of vitamin D levels adds a significant dimension to the research. By exploring the association between vitamin D and brain changes, the study highlights a modifiable factor that could influence disease outcomes. This focus on a potentially modifiable risk factor is particularly valuable for developing preventive or therapeutic strategies aimed at improving the quality of life for PD patients.

Additionally, we acknowledged the study has several limitations. Firstly, as a cross-sectional study, it cannot establish causality. Whether low vitamin D levels directly cause structural changes in the SFG and cognitive decline, or whether other mediating factors are involved, remains to be answered by longitudinal studies and mechanistic research. Secondly, the sample size in this study is relatively small, mainly due to the strict inclusion criteria and the availability of eligible PD patients who completed all imaging and serum assessments. While this sample size is considered sufficient to detect meaningful group differences with acceptable statistical power, based on the exploratory nature of the study and the medium effect sizes observed in our previous research, future studies should incorporate larger sample sizes for further validation. Lastly, the lack of replication and external validation in this study limits the generalizability of our results, highlighting the need for future studies with replication samples and validation groups.

## 5 Conclusion

In conclusion, this study found a significant correlation between GMV reduction in the SFG and vitamin D levels in PD patients, suggesting that vitamin D may be linked to cognitive impairment in PD. Through its anti-inflammatory, antioxidant, and neurotrophic effects, vitamin D might help protect neurons and maintain the integrity of brain structure and function. Future research should further explore the mechanisms of vitamin D’s impact on cognitive function in PD patients and whether vitamin D supplementation could be a potential therapeutic strategy to improve cognitive function in PD patients. By deeply investigating the role of vitamin D in neurodegenerative diseases, new insights and methods for the prevention and treatment of PD and other related diseases can be provided.

## Data Availability

The original contributions presented in the study are included in the article/Supplementary material, further inquiries can be directed to the corresponding authors.

## Footnotes

^1^ https://fsl.fmrib.ox.ac.uk/fsl/fslwiki

^2^ https://www.fil.ion.ucl.ac.uk/spm/

## Supplementary material

SUPPLEMENTARY FIGURE 1 The alternation of gray matter volume in PD. Gray matter atrophy patterns in the PD compared with the HC group and with each other. The PD subgroup showed significantly diffuse GMV loss compared with the HCs group. (TFCE corrected *p* < 0.05).

## Glossary

AD: Alzheimer’s disease
DMN: Default-mode network
FC: Functional connectivity
fc-CC: Functional connectivity correlation coefficient
GMV: Gray matter volume
HCs: Healthy controls
LEDD: Levodopa Equivalent Daily Dose
MMSE: Mini-Mental State Examination
MFG: Middle frontal gyrus
PD: Parkinson’s disease
PD-CI: Parkinson’s disease with cognitive impairment
PD-NC: Parkinson’s disease with normal cognition
PDQ39: Parkinson’s Disease Questionnaire scores
ROIs: Regions of interest
rs-fMRI: Resting-state functional MRI
SFG: Superior frontal gyrus
SFGmed: The medial of superior frontal gyrus
SFGmorb: Medial orbital of superior frontal gyrus
UPDRS: Unified Parkinson’s Disease Rating Scale
VBM: Voxel-based morphometry
VDR: Vitamin D receptors
