# Supplementary figures and images for: Vitamin D-linked vulnerability and functional connectivity alterations in the superior frontal gyrus contributing to cognitive impairment in Parkinson’s disease
Source: Front Aging Neurosci. 2025 Nov 19;17:1657723. doi: 10.3389/fnagi.2025.1657723 (PMC12673341; doi:10.3389/fnagi.2025.1657723)

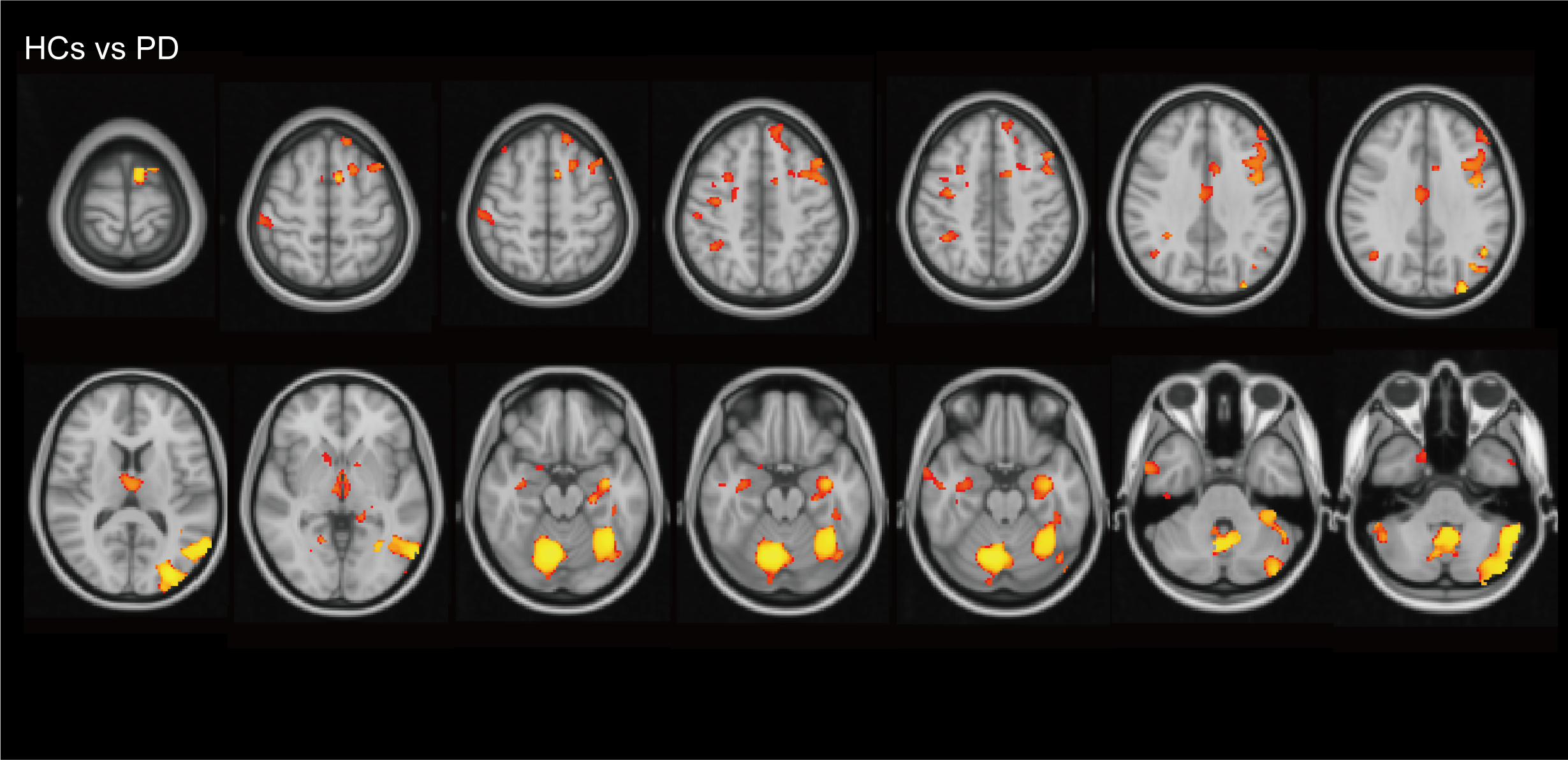

Supplement: SUPPLEMENTARY FIGURE 1 — The alternation of gray matter volume in PD. Gray matter atrophy patterns in the PD compared with the HC group and with each other. The PD subgroup showed significantly diffuse GMV loss compared with the HCs group. (TFCE corrected p < 0.05). [file Image_1.TIF]
