# Supplementary material for: Vitamin D-linked vulnerability and functional connectivity alterations in the superior frontal gyrus contributing to cognitive impairment in Parkinson’s disease
Source: Front Aging Neurosci. 2025 Nov 19;17:1657723. doi: 10.3389/fnagi.2025.1657723 (PMC12673341; doi:10.3389/fnagi.2025.1657723)
Supplement: Supplementary file 2 [file Table_1.DOCX]

Supplementary Table 1 Logistic Regression Analysis of the Impact of SFG Functional Network and Vitamin D Levels on Cognitive Impairment in PD

| fc-CC values of SFG | B | Wald | *P*-value | OR | 95%CI |
| --- | --- | --- | --- | --- | --- |
| Model 1 |  |  |  |  |  |
| SFGmed.L Cluster 2 | -27.125 | 4.939 | 0.026 | 0.000 | 0.000,0.041 |
| SFGmed.R Cluster 2 | 15.871 | 2.328 | 0.127 | 7810730.713 | 0.011,5.582E+15 |
| SFGmorb.R Cluster 1 | -0.269 | 0.000 | 0.983 | 0.764 | 0.000,3.788E+10 |
| SFGmorb.R Cluster 2 | 5.745 | 0.313 | 0.576 | 312.681 | 0.000,1.717E+11 |
| 25(OH)D_3_ | -0.120 | 2.636 | 0.104 | 0.887 | 0.767,1.025 |
| Model 2 |  |  |  |  |  |
| SFGmed.L Cluster 2 | -24.253 | 4.205 | 0.040 | 0.000 | 0.000,0.342 |
| SFGmed.R Cluster 2 | 16.460 | 2.549 | 0.110 | 14081125.264 | 0.024,8.394E+15 |
| SFGmorb.R Cluster 1 | 2.652 | 0.037 | 0.847 | 14.189 | 0.000,7.513E+12 |
| SFGmorb.R Cluster 2 | 6.364 | 0.334 | 0.563 | 580.790 | 0.000,1.362E+12 |
| 25(OH)D_3_ | -0.137 | 3.287 | 0.070 | 0.872 | 0.752,1.011 |
| Age | 0.077 | 2.539 | 0.111 | 1.080 | 0.983,1.186 |
| Model 3 |  |  |  |  |  |
| SFGmed.L Cluster 2 | -22.817 | 3.685 | 0.055 | 0.000 | 0.000,1.617 |
| SFGmed.R Cluster 2 | 18.673 | 2.933 | 0.087 | 128715517.610 | 0.067,2.461E+17 |
| SFGmorb.R Cluster 1 | 3.095 | 0.046 | 0.830 | 22.089 | 0.000,4.370E+12 |
| SFGmorb.R Cluster 2 | 7.350 | 0.411 | 0.522 | 1555.995 | 0.000,8.972E+12 |
| 25(OH)D_3_ | -0.173 | 3.936 | 0.047 | 0.841 | 0.709,0.998 |
| Age | 0.078 | 2.752 | 0.097 | 1.081 | 0.986,1.185 |
| H&Ystage | 0.819 | 1.042 | 0.307 | 2.269 | 0.471,10.943 |

fc-CC, functional connectivity correlation coefficient; PD, Parkinson’s disease；L, left; R, right; SFG, superior frontal gyrus; SFGmed, the medial of superior frontal gyrus; SFGmorb, medial orbita of superior frontal gyrus.
